# B Cell IL-4 Drives Th2 Responses *In Vivo*, Ameliorates Allograft Rejection, and Promotes Allergic Airway Disease

**DOI:** 10.3389/fimmu.2022.762390

**Published:** 2022-03-14

**Authors:** Zhixing Song, Wenjia Yuan, Leting Zheng, Xingan Wang, Vijay K. Kuchroo, Kanishka Mohib, David M. Rothstein

**Affiliations:** ^1^Thomas E. Starzl Transplantation Institute, University of Pittsburgh School of Medicine, Pittsburgh, PA, United States; ^2^School of Medicine, Tsinghua University, Beijing, China; ^3^Department of Kidney Transplantation and Department of Organ Transplantation and General Surgery, Second Xiangya Hospital of Central South University, Changsha, China; ^4^Department of Rheumatology and Clinical Immunology, First Affiliated Hospital of Guangxi Medical University, Nanning, China; ^5^Department of Medicine, University of Pittsburgh Medical Center, Pittsburgh, PA, United States; ^6^Evergrande Center for Immunologic Diseases, Harvard Medical School and Brigham and Women’s Hospital, Boston, MA, United States; ^7^Klarman Cell Observatory, Broad Institute of MIT and Harvard, Cambridge, MA, United States; ^8^Department of Immunology, University of Pittsburgh, Pittsburgh, PA, United States

**Keywords:** B cell, IL-4, transplantation, allergic airway disease (AAD), Th2 (type-2) immune responses

## Abstract

B cells can be polarized to express various cytokines. The roles of IFNγ and IL-10, expressed respectively by B effector 1 (Be1) and Bregs, have been established in pathogen clearance, tumor growth, autoimmunity and allograft rejection. However, the *in vivo* role of B cell IL-4, produced by Be2 cells, remains to be established. We developed B-IL-4/13 iKO mice carrying a tamoxifen-inducible B cell-specific deletion of IL-4 and IL-13. After alloimmunization, B-IL-4/13 iKO mice exhibited decreased IL-4^+^ Th2 cells and IL-10^+^ Bregs without impact on Th1, Tregs, or CD8 T cell responses. B-IL-4/13 iKO mice rejected islet allografts more rapidly, even when treated with tolerogenic anti-TIM-1 mAb. In ovalbumin-induced allergic airway disease (AAD), B-IL-4/13 iKO mice had reduced inflammatory cells in BAL, and preserved lung histology with markedly decreased infiltration by IL-4^+^ and IL-5^+^ CD4^+^ T cells. Hence, B cell IL-4 is a major driver of Th2 responses *in vivo* which promotes allograft survival, and conversely, worsens AAD.

## Introduction

While B cells are central to humoral immunity, they also play an important role shaping the immune response by presenting antigen, providing T cell co-stimulation, and producing various cytokines (1–9). For example, B cell Lymphotoxin-α is required for follicular dendritic cell differentiation and the generation of B cell follicles (10, 11). Regulatory B cells (Bregs) expressing IL-10 and other inhibitory cytokines, play a critical role maintaining self-tolerance, and can inhibit autoimmunity, allograft and tumor rejection, and anti-microbial responses (3, 9, 12–16). Lund and colleagues showed that Th1 cells, IFNγ, or IL-12, can polarize B cells (termed Be1) to express pro-inflammatory cytokines such as IFNγ and IL-12 (5). In contrast, interaction with Th2 cells or IL-4, polarized B cells (termed Be2) to produce IL-2 and IL-4. Be1 and Be2 cells could then promote the *in vitro* differentiation of Th1 and Th2 cells, respectively (5, 17). Subsequently, B cell IFNγ was shown to play an important role in Th1 responses that promote allograft and tumor rejection, autoimmune arthritis, and antibacterial responses (8, 9, 18, 19). In contrast, the role of B cell IL-4 in promoting Th2 responses modulating disease *in vivo*, remains uncertain. Constitutive B cell-specific deletion of IL-4Rα reduced Th2 and increased Th1 responses, enhancing susceptibility to *Schistosoma mansoni* (controlled by Type 2 responses), while reducing Th2-driven cutaneous *Leishmania major* infection and house dust mite (HDM)-induced asthma (20, 21). These studies establish a clear role for IL-4-responsive B cells *in vivo*. However, the role of B cell IL-4 expression itself is less clear. In a bone marrow chimera model, loss of B cell IL-4 expression reduced Th2 and augmented Th1 responses, conferring protection to cutaneous *L. major* (20). However, in HDM-induced asthma, transfer of IL-4/IL-13^−/−^ B cells into μMT mice reduced airway hyperresponsiveness but had no effect on the Th2 response or airway inflammation—suggesting that IL-4 responsiveness, rather than IL-4 production by B cells, drives Th2 responses and airway inflammation (21). In agreement, while IL-4-responsive B cells are required, B cell IL-2 and not IL-4, drove protective Th2 responses to *H. polgyrus* infection (22). Similarly, B cells, but not B cell IL-4, were required for Th2 responses to *Nippostrongylus brasiliensis* infection (7). Thus, IL-4-responsive B cells play an important role in parasitic and asthma immune responses, but the role of B cell IL-4 per se, particularly in Type 2-mediated diseases, remains unclear.

Contradictory outcomes might result from examination of different disease models, but also from different (non-physiologic) approaches used to generate mice with IL-4-deficient B cells. In both cell transfer and bone marrow chimera approaches, B cells develop in constitutively cytokine-deficient environments which might lead to compensatory changes. Given the major role of other B cell cytokines in wide-ranging immune responses, we aimed to establish whether B cell IL-4 is an important driver of *in vivo* Th2 responses using mice with a novel inducible, B cell-specific deletion of IL-4 and IL-13. IL-13 is a signature type 2 cytokine encoded within the Th2 cytokine locus that is coordinately expressed with IL-4 in Th2 cells, while the IL-13 receptor (IL-4Rα:IL-13Rα heterodimer), is largely confined to non-hematopoietic cells (23). Unlike the models described above, these mice are developmentally and physiologically normal until tamoxifen treatment which induces nuclear translocation of Cre which is specifically expressed in B cells. Testing the immune responses in both type I and type II-driven disease models, we determined that B cell IL-4 plays an important role driving Th2 responses *in vivo*, leading to exacerbation of airway inflammation in allergic airway disease (AAD; Th2-driven), and reducing islet allograft rejection (where Th2 responses may be protective). Thus, B cell IL-4 production has a major impact on Th2 cells and amplifies Type 2 immune responses.

## Materials and Methods

### Mice

C57BL/6 (B6), BALB/c and IL-4/IL-13fl/fl mice (BALB/c) were purchased from the Jackson Laboratories. hCD20-ERT2.Cre^+/−^ (BALB/c; kindly provided by Mark Shlomchik, University of Pittsburgh) were used as Cre-controls (24). We crossed hCD20-ERT2.Cre and IL-4/IL-13fl/fl mice to generate hCD20-ERT2.Cre^+/−^ IL-4/IL-13fl/fl mice (B-IL-4/13 iKO). B IL-4/13 iKO and hCD20.Cre-control mice were placed on a 1:1 mixture of normal and tamoxifen-containing chow (Envigo RMS LLC; 250 mg/kg) for 7 days to acclimate, then placed on tamoxifen chow alone (~40 mg intake/d/mouse) for 7 days prior to initiating experiments. Mice remained on tamoxifen chow for the duration of each experiment. Mice were used between 8 and 12 weeks of age. The University of Pittsburgh Animal Care and Use Committee approved all animal studies.

### Immunization, Cell Stimulation, and Flow Cytometry

Mice were immunized with 30 × 10^6^ mitomycin C-treated allogeneic (C57BL/6) splenocytes intraperitoneally (i.p.) and animals were then sacrificed at the indicated times (3, 25, 26). To determine cytokine expression, splenocytes were cultured in RPMI complete media (10% FBS, 2 mM L-glutamine, 100 U/ml Penicillin/Streptomycin, and 50 μM 2-mercaptoethanol) and stimulated for 5 h, as follows: B cell IL-4: 10 μg/mg LPS, 0.75 μg/ml ionomycin, 50 ng/ml PMA (Sigma Aldrich Co.) and monensin (eBioscience Inc.). B cell IL-10: splenocytes were stimulated as above except ionomycin was used at 50 ng/ml. T cells cytokines: 0.50 μg/ml ionomycin and 50 ng/ml PMA. Cells were stained with Aqua Live-Dead Fixable dye (ThermoFisher Scientific Inc.), washed and incubated with Fc block (anti-CD16/CD32), then stained with anti- CD3, CD4, CD8, Siglec-1 and CD19 fluorochrome conjugates (Fisher Scientific Inc. and BioLegend Inc.). For intracellular staining, cells were fixed and permeabilized using BD Fixation/Permeabilization kit (BD Biosciences Inc.). Cytokines were detected using fluorochrome conjugated anti-IL-4, IL-5, IL-10, and IFN-γ (Fisher Scientific Inc. and BioLegend Inc.). Intracellular staining controls were incubated with relevant isotype/fluorochrome controls. BD LSRII or Fortessa flow cytometers were used to collect the data and FlowJo software was used for data analysis (BD Biosciences Inc.).

### Islet Transplants

B6 pancreata were digested with collagenase V (Sigma Aldrich) for 20 min at 37°C, as we described (3). Digested tissue was layered onto a Ficoll gradient and islets collected from the 11 and 20.5% interface after centrifugation at 1,800 rpm for 10 min at 4°C. Islets were washed and incubated in RPMI complete medium overnight at 37°C in 5% CO_2_. Islets were collected and 250 islets/mouse were placed under the kidney capsule of sex-matched allogeneic recipients with streptozotocin-induced diabetes (blood glucose levels >250 mg/dl) (3). Recipients whose glycemia normalized to <150 mg/dl within 2 days were considered successful. Grafts were considered rejected if blood glucose was >250 mg/dl for two consecutive days. Recipients with normal glycemia beyond 100 days were nephrectomized to demonstrate that euglycemia was due to transplanted islets.

### Allergic Airway Disease and Analysis

Mice were immunized with 40 μg of Ova precipitated in Alum by intraperitoneal injection on days -14 and -7. On day 0, mice received 15 μg of intranasal Ova for 3 consecutive days. Bronchoalveolar lavage (BAL) was carried out by injecting 1ml of RPMI into the lungs through the trachea and recovering the injected media. This procedure was repeated 9 more times. The lungs were washed with total of 10 ml per mouse. Total lung tissue infiltrate was isolated by injecting mice with 5 μg of anti-CD45-FITC mAb (i.v.) to stain intravascular lymphocytes for exclusion (27). After 5 min, mice were perfused with 20 ml of PBS containing 2% heparin through the left ventricle while the right ventricle was cut in order to drain the blood. Lungs were removed, diced and transferred to gentle-MACS tubes containing RPMI medium, 0.25 mg/ml of Liberase TL and 0.20 mg/ml of DNAase I. The tissue was then dissociated with a gentle-MACS tissue disrupter and further digested for 30–40 min at 37°C in 5% CO_2_ according to instructions of the manufacturer. The digested tissue was passed through a 40 μm filter and cell suspension washed with PBS. Cells were prepared for flow cytometry as described above, with the addition of anti-CD45-APC mAb to identify leukocytes within lung tissue.

### Statistical Analyses and Graphing

Statistical analyses and graphing were carried out using GraphPad Prism 9. Data were analyzed using unpaired 2-tailed Student’s t-test, ANOVA, or log-rank (Mantel–Cox) test, as indicated. Differences were considered to be significant at p-values <0.05.

## Results

### Acute Reduction in B Cell IL-4 Expression Decreases IL-10^+^ Bregs and Th2 Polarization

**Figure 1:**
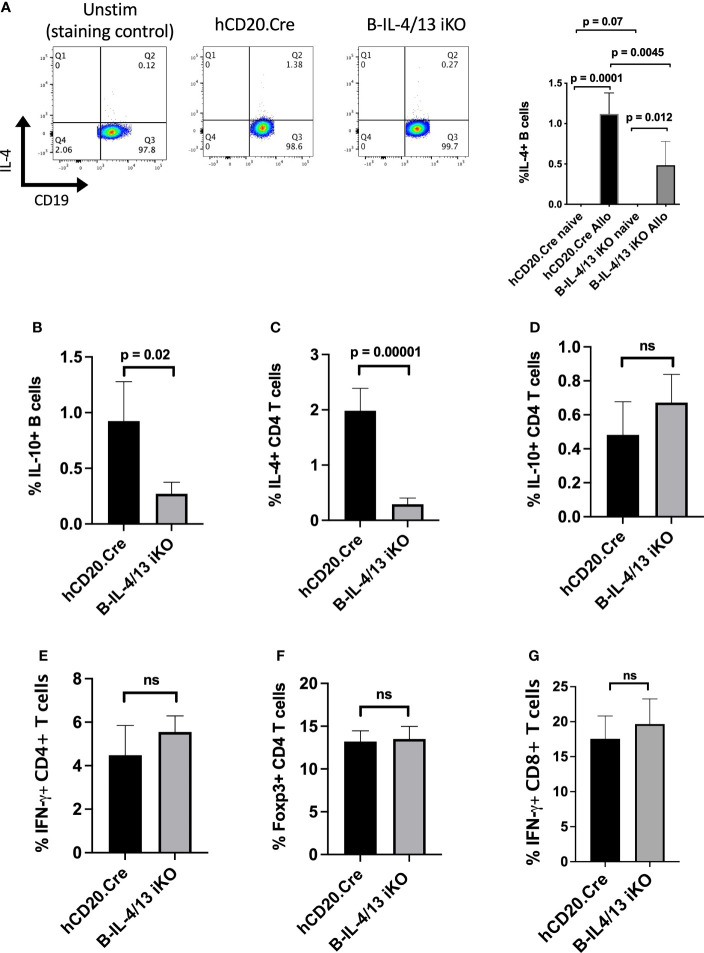
B cell IL-4 expression promotes Breg and Th2 expansion in response to alloantigen challenge. Splenocytes were isolated from Tam-treated hCD20.Cre-control and B-IL-4/13 iKO mice that were unimmunized (naive) or 14 days after alloimmunization (Allo). B cells were stimulated *in vitro* with PMA, ionomycin, LPS and monensin (LPIM) for 5 h. **(A)** Representative FACs plots showing frequency of IL-4 expression on CD19^+^ B cells from unstimulated alloimmunized hCD20.Cre-control mice (negative staining control), and LPIM-stimulated B cells from alloimmunized hCD20.Cre-control and B-IL-4/13 iKO mice. Right Panel: Bar graph shows cumulative data comparing IL-4 expression in B cells from naive vs. alloimmunized hCD20.Cre-control and B-IL-4/13 iKO mice. (IL-4 expression was not detected in naïve mice). **(B–G)** Bar graphs comparing cells from alloimmunized hCD20.Cre-control and B-IL-4/13 iKO mice: **(B)** B cell IL-10 frequency. **(C)** CD4 T cell IL-4 frequency. **(D)** CD4 T cell IL-10 frequency. **(E)** CD4 T cell IFNγ frequency and **(F)** CD4 T cell Foxp3 frequency. **(G)** CD8 T cell IFN-γ frequency. n = 4–6 mice per group in 3 independent experiments. Graphs show mean ± SD. An unpaired Student’s t-test was used to compare differences between the indicated groups. Numbers above each panel signify p-values where significant. ns, not significant.

To examine the role of B cell IL-4 on Th2 differentiation and Th2-mediated immune responses, we crossed hCD20-ERT2.Cre and IL-4/IL-13fl/fl BALB/c mice to generate mice with a tamoxifen (Tam)-inducible B cell-specific deletion of IL-4 and IL-13 (B-IL-4/13 iKO). Mice were placed on tamoxifen chow for two weeks prior to initiating experiments. At baseline, naïve Tam-treated B-IL-4/13 iKO and hCD20.Cre-control mice did not have any significant differences in splenic CD3^+^, CD4^+^, or CD8^+^ T cells or B cell frequency or number (**Supplemental Figures 1A, B**). Further, there were no differences in cytokine expression by CD4^+^ T cells or B cells (**Supplemental Figures 1C, D**). Consistent with previous findings (28), IL-4 expression is essentially undetectable in B cells from naive hCD20.Cre-control or B-IL-4/13 iKO mice (**Supplemental Figure 1D**). To evoke an immune response, Tam-treated B-IL-4/13 iKO and hCD20.Cre-control mice were alloimmunized and spleens harvested on day 14. After alloimmunization, a small but reproducible fraction of B cells expresses IL-4 (**Figure 1A**). B cells from B-IL-4/13 iKO mice had a 60% decrease in frequency of IL-4 expression compared to those from hCD20.Cre-control B cells (**Figure 1A**). Of note, we did not detect IL-13 protein expression by B cells from alloimmunized B-IL-4/13 iKO or hCD20.Cre-control mice (**Supplemental Figure 2A**).

We previously demonstrated that TIM-1^+^ B cells, a population with potent Breg activity, were highly enriched for both IL-4 and IL-10 expression (3). Moreover, B cells from constitutive IL-4^−/−^ and IL-4Rα^−/−^ mice exhibited markedly decreased expression of both TIM-1 and IL-10, suggesting that IL-4 signaling was important for Breg development. In alloantigen immunized B-IL-4/13 iKO mice, acute reduction in B cell IL-4 expression resulted in an ~4-fold decrease in the frequency of IL-10^+^ B cells (Bregs) compared to hCD20.Cre-controls (**Figure 1B** and **Supplemental Figure 2A**). Because the number of B and T cells in spleens of control and iKO mice are comparable (**Supplemental Figure 1A**), these changes in frequency of B and T cell subsets reflect changes in overall number. Thus, acutely reducing endogenous B cell-derived IL-4 markedly reduces Breg IL-10 expression. Examination of CD4^+^ T cells from the same mice revealed that reduced B cell IL-4 resulted in a 7.5-fold decrease in frequency of IL-4 expression, while the expression of IL-10, IFNγ, and Foxp3 were unchanged (**Figures 1C–F** and **Supplemental Figure 2**). Moreover, there was no difference in IFNγ expression by CD8 T cells (**Figure 1G**). Thus, reduction in B cell IL-4 results in a specific decrease in IL-10^+^ Bregs and Th2 cells. This indicates that B cell IL-4 normally promotes Th2-polarization in response to alloimmunization.

### Acute Reduction in B Cell IL-4 Expression Reduces IL-4 Expression by CD4 T Cells and Promotes Allograft Rejection

**Figure 2:**
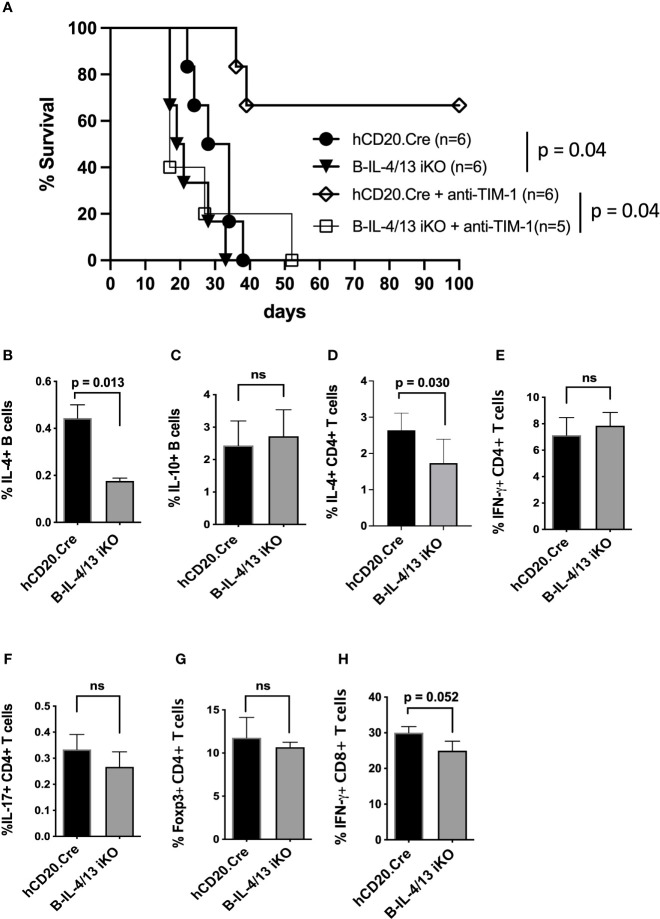
B cell IL-4 expression promotes islet allograft survival. **(A)** Kaplan–Meier plot showing islet allograft graft survival in hCD20.Cre-control and B-IL-4/13 iKO mice that were untreated or treated with anti-TIM-1 (as designated). Log-rank (Mantel–Cox) test was used to compare indicated groups. **(B–H)** Intracellular cytokine and Foxp3 expression by splenic B cells **(B, C)**, CD4^+^ T cells **(D–G)** and CD8^+^ T cells **(H)** from alloimmunized anti-TIM-1 treated hCD20.Cre-control and B-IL-4/13 iKO mice after *in vitro* stimulation, as described in **Figure 1**. n = 4–6 mice per group and in 2 independent experiments. Graphs show mean ± SD. An unpaired Student’s t-test was used to compare differences between the indicated groups. ns, not significant.

Studies performed several decades ago suggested that allograft rejection is exacerbated by Th1 immune responses while Th2 responses promote allograft acceptance (29–34). Given that B cell IL-4 supports Th2 responses after alloimmunization, we examined its role in allograft survival. To accomplish this, diabetic Tam-treated B-IL-4/13 iKO versus hCD20.Cre-control mice were transplanted with fully MHC-mismatched islet allografts. B-IL-4/13 iKO transplant recipients exhibited accelerated rejection of allografts compared to hCD20.Cre-control mice (MST = 21 days vs. 31 days, respectively; p = 0.04, **Figure 2A**). This suggests that acute rejection is exacerbated by reduced B cell IL-4 and resultant decreased Th2 responses in B-IL-4/13 iKO mice, at least when combined with Breg deficiency. In contrast, we previously showed deficiency of either Bregs or B cell IL-10 alone, do not accelerate rejection in untreated recipients (9, 35).

Treatment of islet allograft recipients with tolerogenic anti-TIM-1 mAb (RMT1-10) was shown to increase IL-10 expression by B cells ~3–5 fold and prolongation of allograft survival was dependent on B cell IL-10 expression (3, 9, 36, 37). To determine whether anti-TIM-1 remains effective in the face of reduced B cell IL-4, diabetic Tam-treated B-IL-4/13 iKO and hCD20.Cre-control mice received islet transplants as above but were treated with anti-TIM-1. Anti-TIM-1 markedly prolonged allograft survival in hCD20.Cre-control mice, resulting in long-term survival in 66% of recipients (MST 17days vs. >100 days, respectively; p = 0.04, **Figure 2A**). In contrast, anti-TIM-1 did not prolong allograft survival of B-IL-4/13 iKO mice. After anti-TIM-1 treatment, alloimmunized B-IL-4/13 iKO mice maintained an ~60% reduction in splenic IL-4^+^ B cells compared to control hCD20.Cre mice (**Figure 2B**). However, in this setting, anti-TIM-1 elicited a similar increase in splenic IL-10^+^ Bregs in both strains (**Figures 2B, C**). This contrasts with the decrease in B cell IL-10 expression in alloimmunized B-IL-4/13 iKO mice in the absence of anti-TIM-1 (**Figure 1B**). Regardless, the frequency of splenic IL-4^+^ CD4 T cells was still reduced in anti-TIM-1 treated B-IL-4/13 iKO recipients compared to hCD20.Cre-control mice, while the frequency of IFNγ, IL-17 and Foxp3 expressing CD4 T cells and IFNγ expressing CD8 T cell remained unchanged (**Figures 2D–H**). These data support the notion that B cell IL-4 supports Th2 differentiation and that B cell IL-4 and Th2 polarization play an important role in anti-TIM-1 mediated allograft survival that is independent from the role of IL-10^+^ Bregs, Tregs or Th1 responses.

### Acute Reduction in B Cell IL-4 Expression Reduces Th2 Expression and Markedly Inhibits Allergic Airway Disease (AAD)

**Figure 3:**
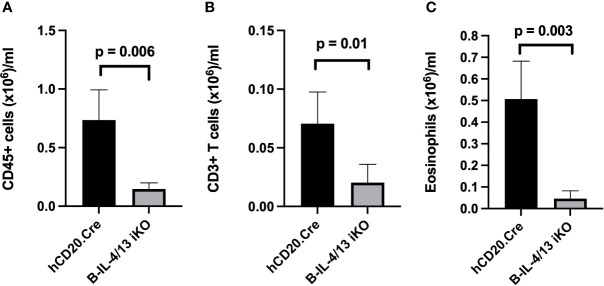
B cell IL-4 expression promotes airway inflammation in AAD. BAL from Ova-sensitized hCD20.Cre-control and B-IL-4/13 iKO mice was examined for **(A)** Total CD45^+^ cells; **(B)** Total CD3^+^ T cells; and **(C)** Eosinophils. n = 3–4 mice/group in 2 independent experiments. Graphs show number of cells/ml of lavage fluid expressed as mean ± SD. An unpaired Student’s t-test was used to compare differences between the indicated groups.

IL-4 expression by Th2 cells is a major contributing factor to pathophysiology of AAD including airway inflammation (38, 39). To directly address the role of B cell IL-4 expression on Th2 polarization and airway inflammation, we subjected Tam-treated B-IL-4/13 iKO and hCD20.Cre-control mice to Ova-induced AAD. In naïve mice, ~120,000 CD45^+^ leukocytes were recovered in BAL from either hCD20.Cre-control or B-IL-4/13 iKO mice (**Supplemental Figure 3A**). After induction of AAD, there was virtually no increase in number of CD45^+^ leukocytes in the bronchoalveolar space of B-IL-4/13 iKO mice, whereas those from hCD20.Cre-control mice increased ~5-fold (**Figure 3A**). BAL fluid had 3.5× more CD3 T cell and 12.5× more eosinophils in hCD20.Cre-controls (**Figures 3B, C**). Very few, if any, B cells were found in the BAL of either mouse strain (data not shown).

**Figure 4:**
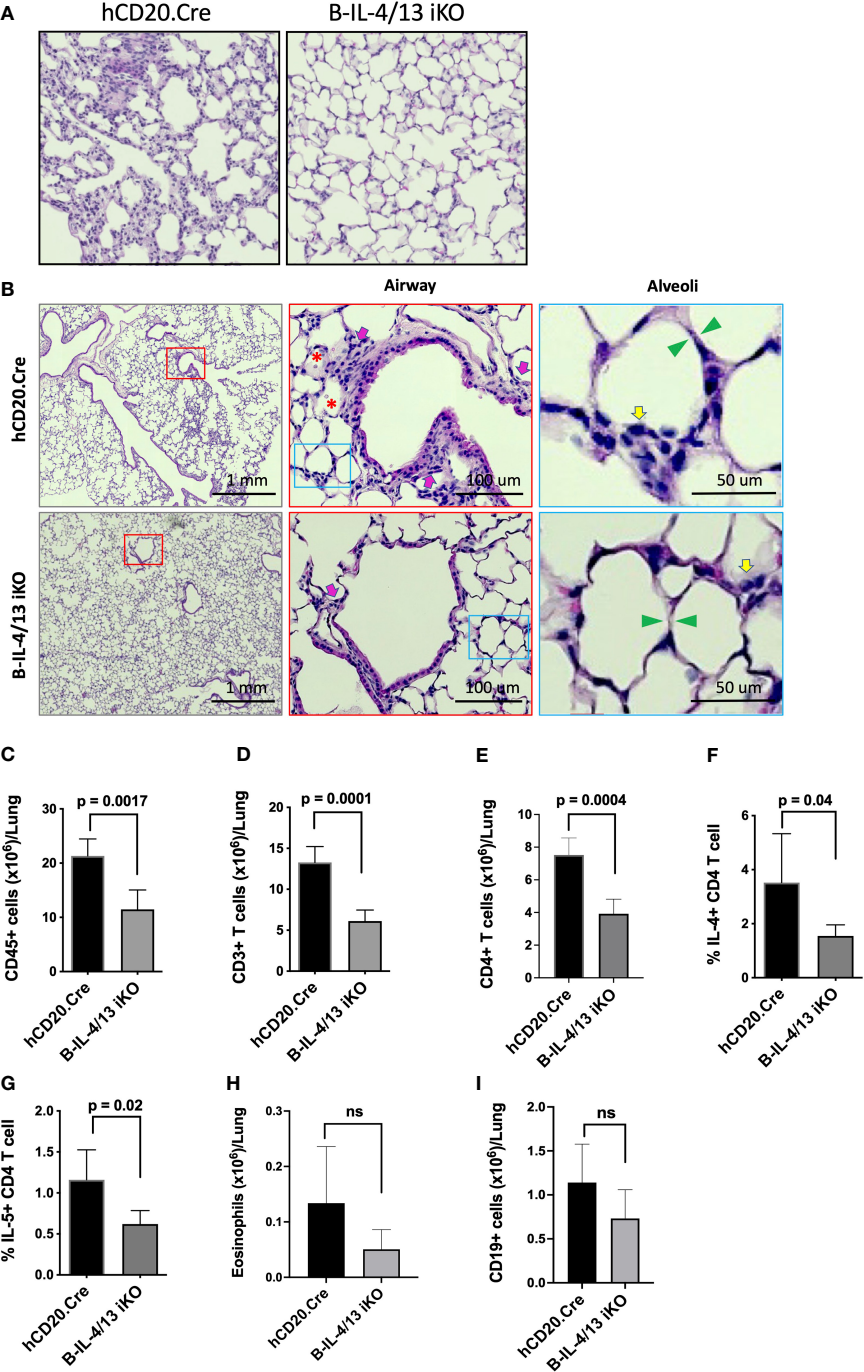
B cell IL-4 expression promotes lung tissue inflammatory cell infiltration and Th2 polarization in AAD. Lungs from Ova-sensitized hCD20.Cre-control and B-IL-4/13 iKO mice were harvested and either paraffin embedded or digested to examine immune cell infiltration. **(A)** Representative lung tissue sections stained with H&E showing degree of immune infiltrate in hCD20.Cre-control and B-IL-4/13 iKO lungs (×20 magnification). **(B)** Left panels: Large area scan image that was stitched together (×10 magnification) of H&E stained lungs with red boxes highlighting representative small airways shown at higher magnification in middle panels. Middle Panels: Red box inset image of area surrounding small airways. Pink arrows indicate inflammatory cells around airways. Red asterisks indicate pink proteinaceous debris and red blood cells. Blue boxes show alveoli in area of airways, further magnified in right panels. Right Panels: inset image of alveoli from airway image. Yellow arrows indicate inflammatory cells in alveolar/interstitial spaces. Green arrows show alveolar septa. **(C–I)** show quantification of infiltrating leukocyte populations obtained from digested lung tissue. **(C)** Total CD45^+^ cells/lung. **(D)** Total CD3^+^ T cells/lung. **(E)** Total CD4^+^ T cells/lung. **(F)** Frequency of lung IL-4^+^ CD4 T cells. **(G)** Frequency of lung IL-5^+^ CD4 T cells. **(H)** Total Eosinophils/lung. **(I)** Total CD19^+^ B cells/lung. n = 3–5 mice/group in 2 independent experiments. Graphs show mean ± SD. An unpaired Student’s t-test was used to compare differences between the indicated groups. *p < 0.05, **p < 0.001, ***p < 0.0001. ns, not significant.

Lung histopathology in hCD20.Cre-control mice revealed marked thickening and infiltration of inflammatory cells into the alveolar septae compared to B-IL-4/13KO mice, which had preserved lung architecture (**Figures 4A, B**). Moreover, in hCD20.Cre-control mice, an inflammatory cell infiltrate and proteinaceous debris was observed in the alveolar space and an inflammatory infiltrate was observed around a proportion of small airways (**Figure 4B**). In contrast, B-IL-4/13 iKO mice had clear alveolar spaces and no infiltrate around small airways (**Figures 4A, B**). No differences were observed in epithelial thickening between the two groups. To quantitate and characterize the parenchymal infiltrate, lung tissue was digested and intravascular lymphocytes were gated out (see *Materials and Methods*; **Supplemental Figure 4**). As seen in alloimmunized mice, after induction of AAD, no B cell IL-13 could be detected in lung tissue from either B-IL-4/13 iKO or hCD20.Cre control mice (**Supplemental Figure 5**). Naïve Control and B-IL-4/13 iKO mice, had a similarly low number of resident CD45^+^ cells in lung tissue, of which only a small fraction was comprised of T or B lymphocytes or eosinophils (**Figures 4C–E, H, I**). After induction of AAD, lung tissue from hCD20.Cre-control mice exhibited a >5-fold increase in infiltration by (CD45^+^) leukocytes (from 4.9×10^6^ to 21×10^6^), ~75% of which was comprised by a marked expansion of T cells. B cell infiltration increased ~5-fold but still comprised only about 5% of the total infiltrate. Eosinophil numbers increased markedly, but their contribution to the total infiltrate was minor. Despite the preservation of lung architecture and normalization of BAL seen in B-IL-4/13 iKO mice after AAD induction, infiltration of leukocytes and lymphocytes into lung parenchyma was increased compared to baseline levels seen in naive mice (**Figures 4B–F**). However, in comparison to hCD20.Cre control mice, after AAD induction, B-IL-4/13 iKO mice exhibited a 2-fold decrease in the number of infiltrating (CD45^+^) leukocytes, including an ~2-fold decrease in total (CD3^+^) and CD4^+^ T cells (**Figures 4C–E**). Moreover, amongst CD4 T cells in lung tissue, there was an ~2-fold decrease in IL-4 and IL-5 expressing cells in B-IL-4/13 iKO mice (**Figures 4F, G**). Despite the marked differences in BAL, there was no significant difference between B-IL-4/13 iKO and hCD20.Cre control mice in eosinophil infiltration into lung tissue, nor were there differences in B cell infiltration (**Figures 4H, I**). Thus, acute reduction in B cell IL-4 significantly reduced Th2 polarization, lung T cell infiltration, and airway inflammation in AAD.

## Discussion

Despite their relative paucity, B cells expressing various cytokines have a major impact on immune responses in infection, autoimmunity, cancer and transplantation (40–43). *In vitro* studies first showed that Th2 cells (or IL-4) could polarize B cells to produce IL-4, which in turn, amplified Th2 responses (5, 17). Subsequently, it was shown that IL-4-responsive B cells are important for Th2 polarization, and promoted clearance of helminthic infections, while worsening asthma and leishmaniasis (20, 21). However, the *in vivo* role of B cell IL-4 *per se* was unclear, with studies showing that it was required for Th2 responses with resultant worsening of leishmaniasis, but it was not required for Th2 responses and protection from helminthic infections or pulmonary/airway inflammation in allergic asthma (7, 20–22).

To directly address the participation of B cell IL-4 in Th2 responses, we developed a novel mouse strain which is developmentally normal until tamoxifen-mediated acute deletion of B cell IL-4 (B-IL-4/13 iKO). We were unable to detect IL-13 expression by B cells obtained after immunization of hCD20.Cre control mice in either disease model, even after *in vitro* stimulation. Harris et al. did show that MD4 BCR transgenic B cells express low levels of IL-13 mRNA after 3-day culture with antigen and primed Th2 cells followed by PMA and ionomycin for 24 h; however, IL-13 protein was not reported (5). While we cannot eliminate a contribution from loss of IL-13 in our animal model, the lack of B cell IL-13 protein expression in AAD or allograft models suggests that this cytokine does not contribute significantly to our findings. Acute reduction of B cell IL-4 reduces B cell IL-10 expression and reduces Th2 responses, without affecting Th1 or Treg frequency. The latter is interesting since Bregs have generally been shown to correlate with both Foxp3 and IL-10 expression by CD4 cells (3, 9, 44). Our data suggests that neither Breg IL-10 nor IL-4 are required to support Treg/Tr1 subsets. The combination of reduced B cell IL-4 and Th2 cells markedly reduces pulmonary inflammation in AAD. Notably, this occurs despite the reduction in IL-10^+^ Bregs, which in and of themselves are protective in this model (3).

Our findings indicate that IL-4 expression by B cells promotes Th2 responses that underlie allergic diseases such as asthma. While B cells may not produce IL-13, they induce Th2 cells that secrete IL-13 which has pleiotropic effects in asthma pathobiology (45). The current results should prompt a thorough search for IL-4-expressing B cells in human BAL and biopsy material from patients with asthma or other allergic diseases. In this regard, small studies show that B cell depletion with anti-CD20 (rituximab) reduces steroid dependence in asthma associated with eosinophilic granulomatosis with polyangiitis (Churg–Strauss syndrome), and significantly reduces atopic dermatitis where a reduction in IL-13 production, but not IgE, was observed (46, 47). While such insight may not impact ongoing attempts to target IL-4 and IL-13 in treatment of asthma, it could prompt further studies into B cell-directed therapies for allergic disease.

In the allograft setting, studies performed at the advent of the Th1/Th2 paradigm, suggested that Th2 cytokines might promote allograft survival. For example, STAT6^−/−^ mice (Th2-deficient) exhibited reduced survival of minor histocompatibility antigen mismatched cardiac allografts in untreated recipients. Additionally, anti-IL-4 blocked the ability of splenocytes from anti-CD4 treated mice to adoptively transfer tolerance to new allograft recipients and prevented induction of neonatal tolerance (32, 33, 48). None of these approaches is specific for Th2 cells *per se*, and they should now be more broadly viewed as resulting from inhibition of type 2 responses. Ultimately, interest in the role of Th1 versus Th2 cells in allograft survival was overshadowed by other Th subsets and regulatory cells. Here, in the absence of therapy, the combined decrease in IL-10^+^ Bregs and Th2 cells resulting from reduced B cell IL-4, accelerates rejection in otherwise untreated fully MHC-mismatched islet allograft recipients. In comparison, we previously showed that lack of Bregs or B cell IL-10 alone do not hasten rejection (35). More telling, is that deficiency B cell IL-4 prevents induction of long-term allograft survival by anti-TIM-1 despite normal Breg induction in this setting, suggesting that deficiency in B cell IL-4 and resulting decrease in Th2 cells, prevent tolerance. Of course, it remains possible that other compensatory changes resulting from an acute decrease in B cell IL-4 also contribute to the loss of tolerance. Unfortunately, we do not yet have specific agents to target particular subsets of B cells that express harmful versus salutary cytokines. Cases in point, were attempts to use anti-CD20 in the peri-transplant period to limit subsequent antibody-mediated rejection in both renal and cardiac transplantation (49, 50). Unfortunately, both acute cellular renal allograft rejection and cardiac allograft vasculopathy were significantly increased after anti-CD20 treatment, suggesting that B cells play a critical regulatory role early after transplantation. Ultimately, a better understanding of the biology of IL-4-expressing B cells and the timing of their involvement in immune-mediated disease will help us specifically target this B cell subset in attempts to improve patient outcomes. It is interesting that anti-TIM-1 can induce Bregs in this setting while the deficiency in B cell IL-4 is maintained. This provides hope that different subsets of B cells might be individually targeted to inhibit asthma or prevent allograft rejection.

In summary, acute deficiency in B cell IL-4 reduces Th2 polarization indicating that B cell IL-4 is an important driver of Th2 responses *in vivo*. Acute reduction of B cell IL-4 also reduces B cell IL-10 expression, which can be overcome with antibody mediated ligation of TIM-1—a potent inducer of Bregs. As such, a reduction in B cell IL-4 markedly reduces AAD-mediated lung inflammation, while promoting allograft rejection.

## Data Availability Statement

The original contributions presented in the study are included in the article/**Supplementary Material**. Further inquiries can be directed to the corresponding author.

## Ethics Statement

The animal study was reviewed and approved by the University of Pittsburgh Animal Care and Use Committee.

## Author Contributions

DR conceived the study. ZS, WY, LZ, XW, and KM conducted study and contributed to experimental data collection. KM, VK, and DR contributed to experimental design. KM and DR wrote manuscript. All authors listed have made a substantial, direct, and intellectual contribution to the work and approved it for publication.

## Funding

This work was supported by NIH grants R01AI114587 (DMR) and P01129880 (DMR and VKK). KM was supported by the Joseph A. Patrick Research Fellowship in Transplantation.

## Conflict of Interest

The authors declare that the research was conducted in the absence of any commercial or financial relationships that could be construed as a potential conflict of interest.

## Publisher’s Note

All claims expressed in this article are solely those of the authors and do not necessarily represent those of their affiliated organizations, or those of the publisher, the editors and the reviewers. Any product that may be evaluated in this article, or claim that may be made by its manufacturer, is not guaranteed or endorsed by the publisher.
