# Supplementary material for: B Cell IL-4 Drives Th2 Responses In Vivo, Ameliorates Allograft Rejection, and Promotes Allergic Airway Disease
Source: Front Immunol. 2022 Mar 14;13:762390. doi: 10.3389/fimmu.2022.762390 (PMC8963939; doi:10.3389/fimmu.2022.762390)
Supplement: Supplementary file 1 [file DataSheet_1.pdf]

# Supplemental Figure 1

A)

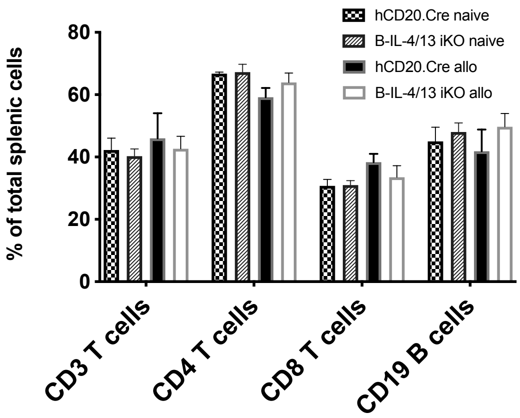


B)

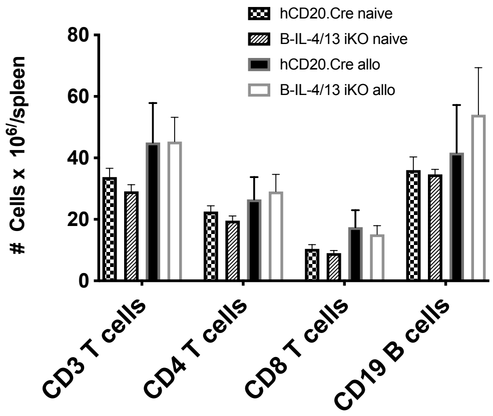


C)

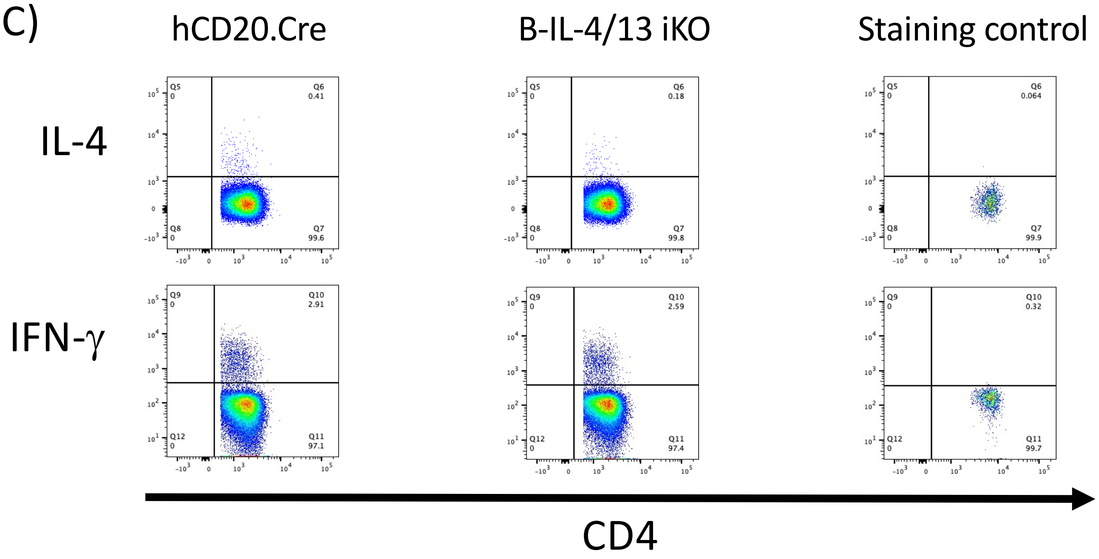


D)

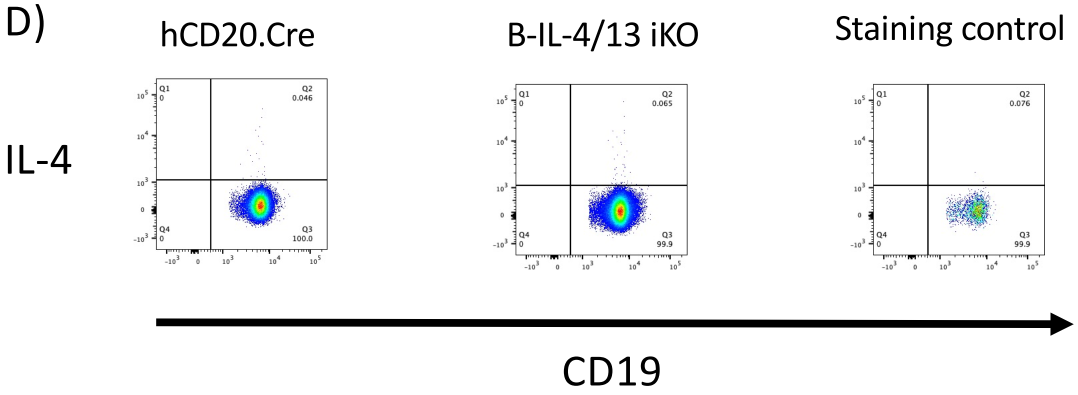


Bar graphs showing frequency **(A)** and cell number **(B)** of total (CD3+), CD4+, and CD8+ T cells and CD19+ B cells in spleens of naïve and allo-immunized Tam-treated hCD20.Cre-control and B-IL-4/13 iKO mice. n= 3-5 mice/group. Graphs show mean ± SD. Representative flow cytometry plots showing cytokine expression by intracellular staining of splenic lymphocytes from naïve hCD20.Cre-control and B-IL-4/13 iKO mice: **(C)** CD4+ T cells stained for IL-4 and IFNγ with corresponding staining controls. **(D)** CD19+ B cells stained for IL-4 with corresponding staining control.

# Supplemental Figure 2

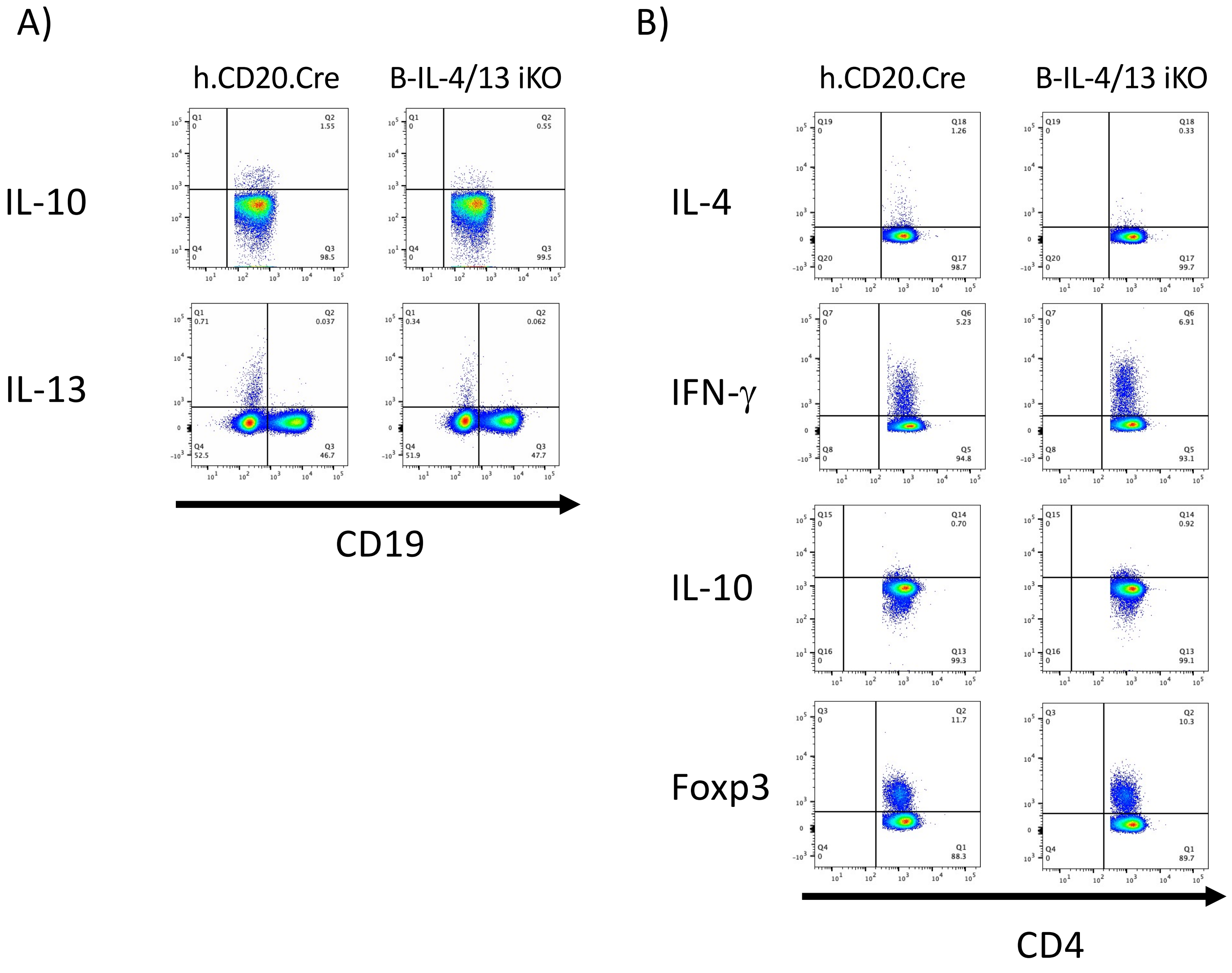


Representative flow cytometry plots showing intracellular staining for cytokines and Foxp3 in splenic B and CD4+ T cells from alloimmunized mice (day 14). (Cumulative data shown in Fig 1). **(A)** CD19+ B cell intracellular IL-10 staining (CD19 gate) and IL-13 staining (lymphocyte gate). **(B)** CD4+ T cell intracellular staining (CD4 gate) for each indicated cytokine or transcription factor.

Supplemental Figure 3

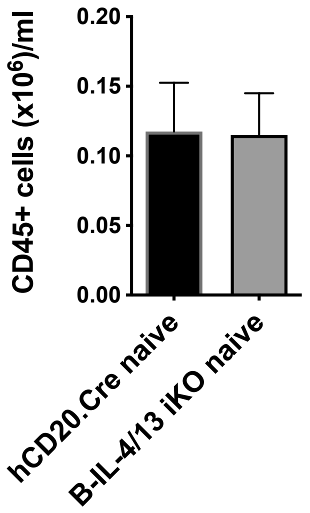


BAL cell numbers recovered from naïve mice from TAM-treated hCD20.Cre-Control and B-IL-4/13 iKO. **A)** Total CD45+ cells recovered. n = 4-5 mice per group.

# Supplemental Figure 4

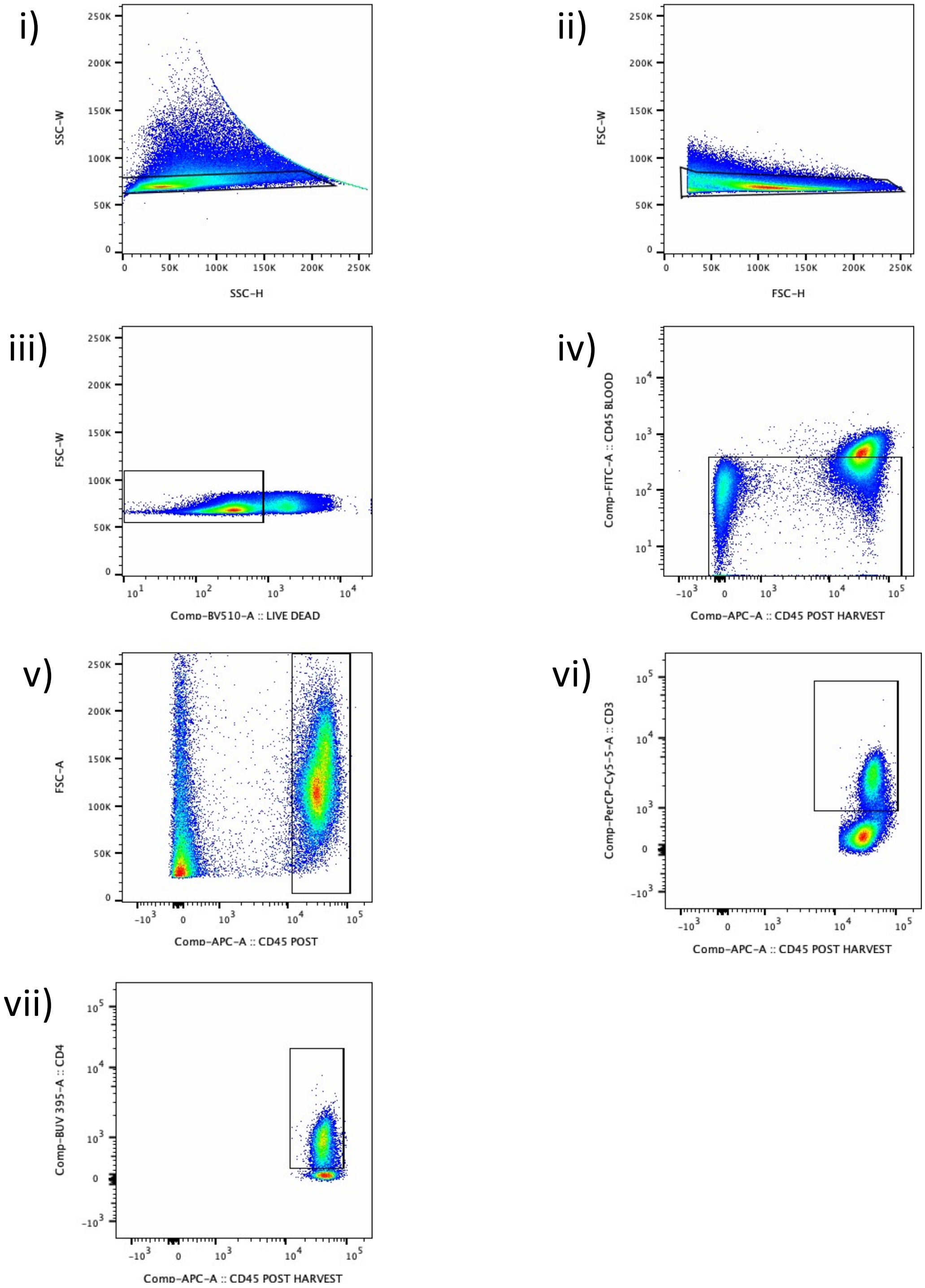


Representative flow cytometry plots showing sequential gating strategy used to examine CD45+ cells infiltrating lung tissue in Figure 4. (i) SSC-W/SSC-H and (ii) FSC-W/FSC-H doublet exclusion, (iii) Dead cell exclusion, (iv) Exclusion of intravascular CD45+ cells (after injection of intravenous CD45-FITC), (v) total tissue CD45+ gate, (vi) CD3+ T cell gate, (vii) CD4+ T cell gate.

# Supplemental Figure 5

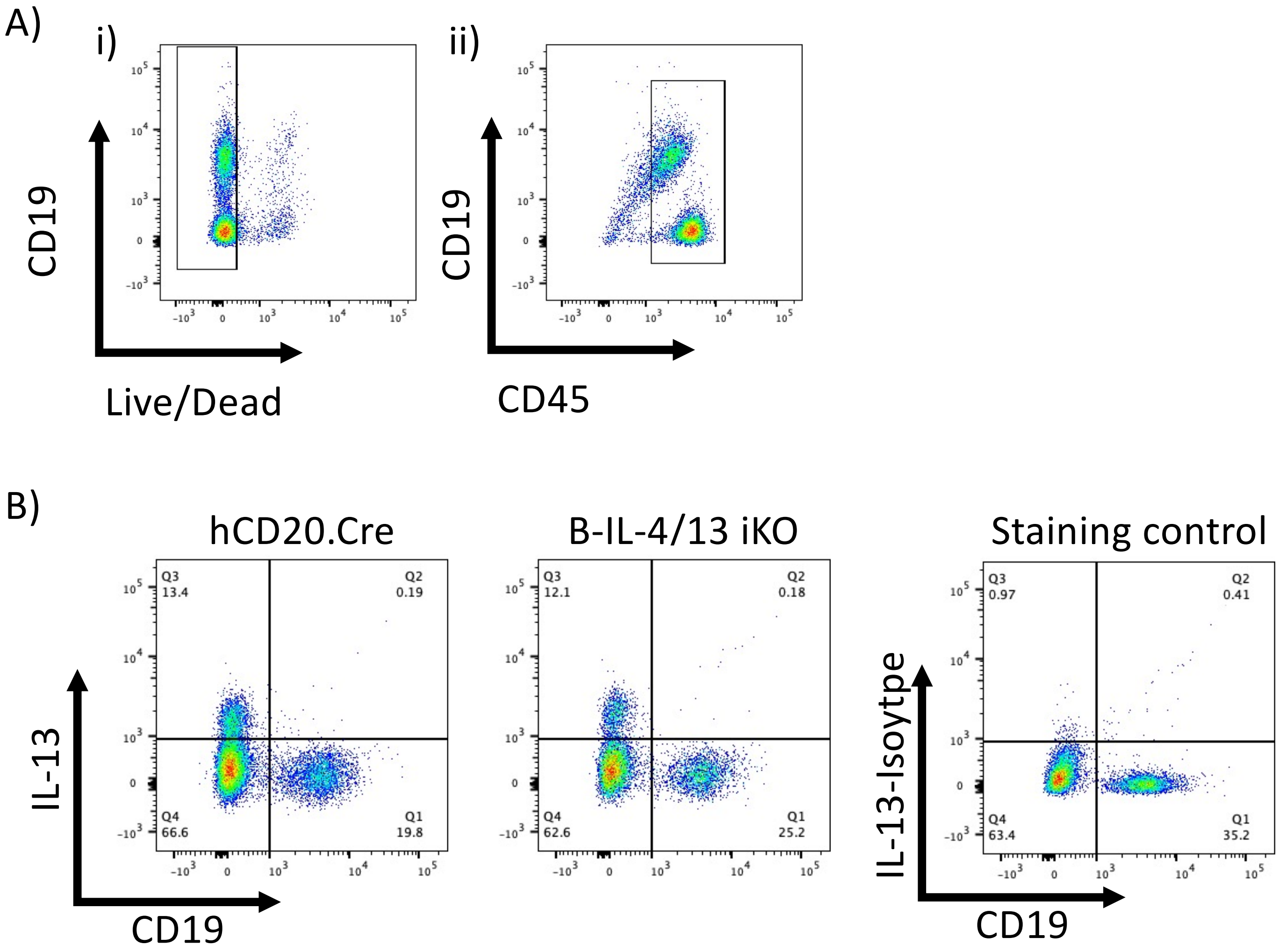


Representative flow cytometry plots showing intracellular staining of IL-13 in B cells in comparison to total lymphocytes from digested lungs of Ova-immunized mice (ADD model). Cells were stimulated in vitro for 5 hours with LPIM. **A)** Gating strategy : i) Dead cell exclusion, ii) CD19+CD45+ total leukocytes gate, **B)** Intracellular staining for IL-13 expression by total CD45+ cells vs. CD19+ B cells isolated from digested lungs of hCD20.Cre-control, B-IL-4/13 iKO and isotype staining control.
